# Nutritional Signaling Regulates Vitellogenin Synthesis and Egg Development through Juvenile Hormone in *Nilaparvata lugens* (Stål)

**DOI:** 10.3390/ijms17030269

**Published:** 2016-02-26

**Authors:** Kai Lu, Xia Chen, Wen-Ting Liu, Xin-Yu Zhang, Ming-Xiao Chen, Qiang Zhou

**Affiliations:** 1 College of Life Sciences, Fujian Agriculture and Forestry University, Fuzhou 350002, China; lukaiqw@163.com (K.L.); 18659105423@163.com (X.C.); liuwting123@163.com (W.-T.L.); 2 State Key Laboratory of Biocontrol, School of Life Sciences, Sun Yat-sen University, Guangzhou 510275, China; 13076721421@163.com (X.-Y.Z.); mingchen119@126.com (M.-X.C.)

**Keywords:** Juvenile hormone, vitellogenesis, nutritional signaling, RNA inteference, *Nilaparvata lugens*

## Abstract

Insect female reproduction which comprises the synthesis of vitellogenein (Vg) in the fat body and its incorporation into developing oocytes, needs a large amount of energy and food resources. Our previous studies found that juvenile hormone (JH) regulates vitellogenesis in the brown planthopper, *Nilaparvata lugens*. Here, we report on the role of JH in nutrient-regulated Vg synthesis and egg development. We first cloned the genes coding for juvenile hormone acid methyltransferase (JHAMT) which is involved in JH biosynthesis and methoprene-tolerant (Met) for JH action. Amino acids (AAs) induced the expression of *jmt*N, while showing no effects on the expression of *met* using an artificial diet culture system. Reduction in JH biosynthesis or its action by RNA interference (RNAi)-mediated silencing of *jmt*N or *met* led to a severe inhibition of AAs-induced Vg synthesis and oocyte maturation, together with lower fecundity. Furthermore, exogenous application of JH III partially restored Vg expression levels in *jmt*N RNAi females. However, JH III application did not rescue Vg synthesis in these *met* RNAi insects. Our results show that AAs induce Vg synthesis in the fat body and egg development in concert with JH biosynthesis in *Nilaparvata lugens* (Stål), rather than through JH action.

## 1. Introduction

Nutrition and reproduction are important physiological processes in insects and nutrients have long been known to play key roles in the regulation of reproduction [1,2]. Egg maturation and successful reproduction requires an input of energy-rich food resources to achieve a nutritional status [3,4]. Vitellogenesis, an important process in insect reproduction, is a nutrient-dependent process. Initiation of vitellogenesis by feeding is a crucial event in the reproductive cycle of the anautogenous insects [5,6]. The free amino acids (AAs) concentration in the hemolymph increases dramatically after a blood meal in *Aedes aegypti* [7] and *Culex pipiens* [8]. Furthermore, AAs derived from the blood meal have been demonstrated to be the key nutritional signals to stimulate *Vg* gene transcription and Vg protein synthesis in the fat body of mosquito [9,10,11]. Although the key role of nutrients in regulation of reproduction has been demonstrated in many insect species, very little is known about its molecular mechanism. Recently, the hormonal control of vitellogenesis has been described in many insects, and hormones are suggested to be involved in nutritionally dependent reproduction [2,12,13].

Juvenile hormone (JH), a sesquiterpenoid hormone synthesized and secreted by the corpus allatum (CA), regulates many aspects of insect reproduction including vitellogenesis, oocyte maturation and ovarian growth [14,15]. JH-regulated vitellogenesis has been reported in different kinds of insects, including Dictyoptera [16], Hymenoptera [17,18], Coleoptera [19], Orthoptera [20] and Hemiptera [21] including mosquitoes [22]. JH has been shown as part of a transduction system that balances nutritional status and controls reproduction in mosquitoes [13,23]. The nutrient-deficient *A. aegypti* developed into smaller adults and showed lower JH titers. Topical application of JH to these small mosquitoes could enable them to complete their first gonotrophic cycle after a single blood meal [24]. In starved adult female *Blattella germanica*, JH synthesis and Vg production are low [6]. Starvation of female *Tribolium castaneum* also led to inhibition of Vg synthesis whereas feeding after starvation stimulated Vg synthesis and egg maturation. However, in the starved beetles where genes coding for protein involved in JH biosynthesis (juvenile hormone acid methyltransferase, JHAMT) was disrupted by RNAi, Vg expression was severely inhibited even after feeding, suggesting that JH is indispensable for the nutrient-dependent Vg synthesis [12]. However, the mechanism of JH action in regulating nutrient-induced vitellogenesis remains obscure.

This report attempts to find out the role of JH III in nutrient-regulated vitellogenesis using the brown planthopper, *Nilaparvata lugens* (Hemiptera), which is an ideal research model to address this question. Additionally, robust RNAi and rapid JH response can be achieved in the adult females [25,26,27]. Using a newly developed artificial diet culture system, we found that nutrients (AAs) are necessary for the expression of *jmt*N which is involved in JH biosynthesis [28,29]. Depletion of *jmt*N and *met* (Methoprene-tolerant, JH receptor) show that JH is required for AAs-induced Vg synthesis, and knockdown of *jmt*N or *met* severely inhibited ovarian development and reduced fecundity. Our results suggest that the JH pathway mediates AAs-induced Vg synthesis in the fat body playing an important role in ovarian growth and development.

## 2. Results

### 2.1. Identification of Genes Involved in JH Signaling and Phylogenetic Analysis

The cDNA sequence of *met* (KJ690934) is 2895 bp with an open reading frame (ORF) of 2391 bp. *Met* ORF encodes a protein of 796 amino acids with a predicted molecular mass of 89 kDa and an isoelectric point (pI) of 5.53. A basic Helix-Loop-Helix (bHLH) domain and two highly conserved Pert-Arnt-Sim (PAS) domains, which were shown to be sufficient for JH binding, were identified in Met. The complete ORF of *jmt*N (KP769805) is 840 bp in length encoding a 279-amino acid polypeptide with a calculated molecular weight of 31.76 kDa and a theoretical pI of 5.46. *N. lugens* JHAMT has a highly conserved *S*-adenosylmethionine (SAM) binding motif IMDVGCGPG [29].

**Figure 1:**
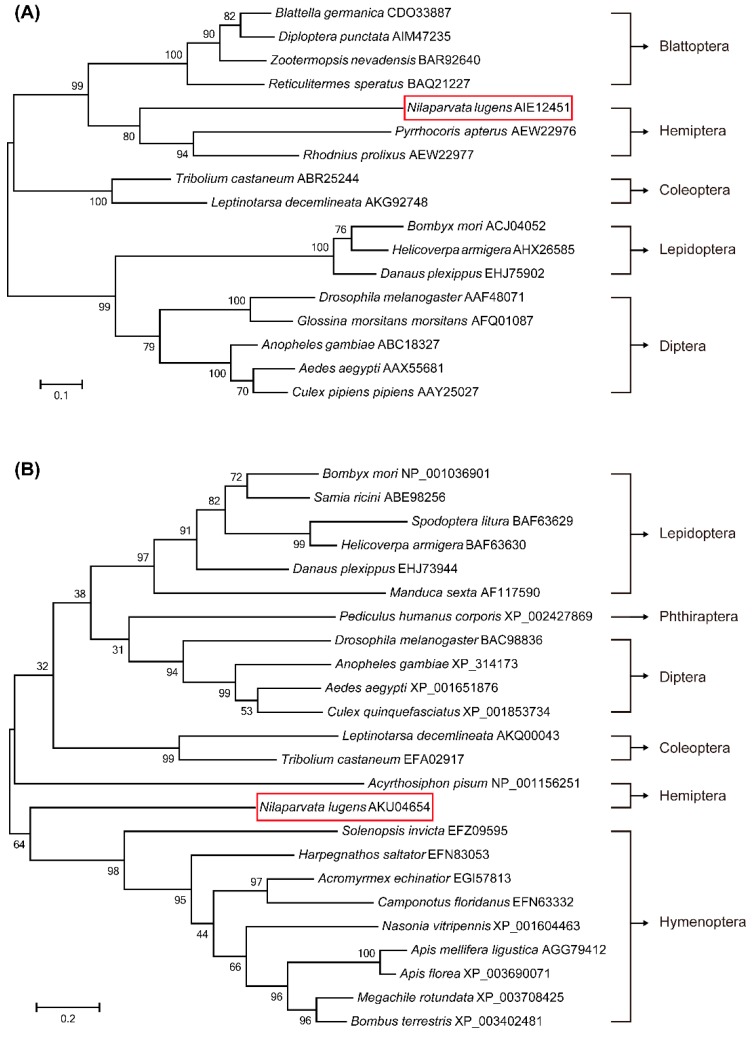
Phylogenetic analysis of Met (**A**) and JHAMT (**B**) from different insect species based on the amino acid sequences alignment. Protein sequences were downloaded from the GenBank database and the accession numbers are shown. The phylogenetic tree was generated by MEGA6 using the Maximum Likelihood method. Bootstrap values (1000 replicates) are shown in the cladogram. The genetic distance is drawn to scale.

To reveal the evolutionary relationship of Met orthologs, a comprehensive phylogenic tree was constructed based on 17 Met protein sequences from five insect orders with 1000 bootstrap replicates. The phylogenetic analysis indicated that *N. lugens* Met is closely related to two orthologs of Hemiptera: *Pyrrhocoris apterus* and *Rhodnius prolixus* (Figure 1A). The phylogenetic relationship of JHAMTs derived from 24 insect species was similarly investigated showing that *N. lugens* JHAMT is a monophyletic branch with a large genetic distance from other insects in the phylogenetic tree. The most closely related protein sequence to JHAMT (34% identity) is from a Hymenoptera insect: *Solenopsis invicta* (EFZ09595) (Figure 1B).

### 2.2. Tissue-Specific Expression Profiles of Met and JmtN Examined by qRT-PCR

**Figure 2:**
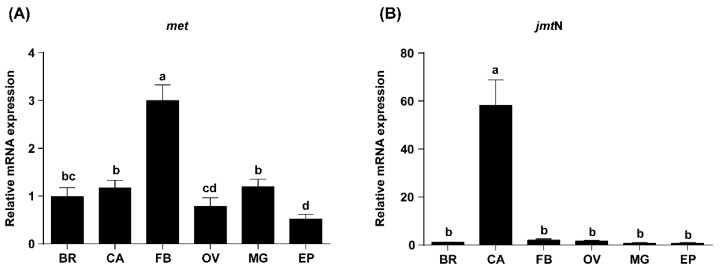
Tissue-specific expression profiles of *met* (**A**) and *jmt*N (**B**). All tissues were dissected from three-day-old adult females. BR: brain; CA: corpora allata; FB: fat body; OV: ovary; MG: Malpighian tubules; EP: epidermis. Values represent the mean of 7–8 independent pools of 30–50 females and three technical replicates, normalized with *β-actin* (*n* = 7–8). Vertical bars indicate standard errors. Different lowercase letters above the columns indicate significant differences within different tissues (one-way ANOVA, *p* < 0.05).

M*et* and *jmt*N mRNA expression levels in the brain, corpora allata, fat body, ovary, Malpighian tubules and epidermis from three-day-old adult females were analyzed by qRT-PCR. *Met* transcripts were detected in all tissues and were primarily expressed in the fat body, with relatively lower expression in the ovary and epidermis (Figure 2A). *Jmt*N mRNA expression was almost exclusively located in the corpora allata. Trace amounts of *jmt*N mRNA were present in the brain, fat body and ovary (Figure 2B). Since expression of *met* was highest in the fat body and transcripts of *jmt*N were mostly produced in the corpora allata, the fat body was used to investigate the changes of *met* mRNA and the corpora allata was used to assess the changes in *jmt*N transcript levels.

### 2.3. AAs Regulate Vg Synthesis in Adult Females

**Figure 3:**
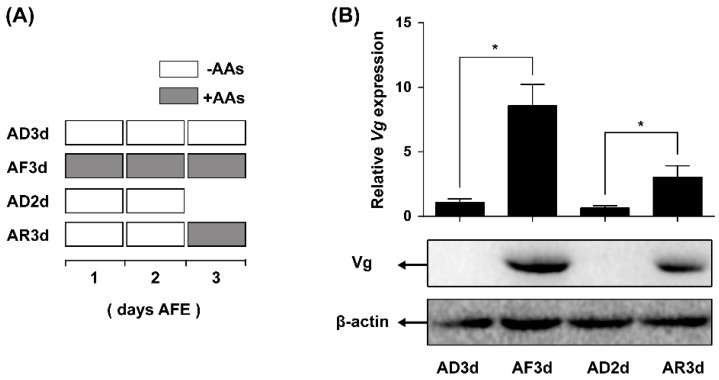
AAs regulate Vg synthesis. (**A**) Diagram of experimental design. AFE, after adult female emergence. AD3d, three-day-old AAs-deprived females; AF3d, three-day-old AAs-fed females; AD2d, two-day-old AAs-deprived females; AR3d, two-day-old AAs-deprived females were fed on normal diet (+AAs) for another day; (**B**) Relative expression levels of *Vg* mRNA in the fat body were detected by qRT-PCR using *β-actin* as a reference. Reactions were performed based on independent RNA sample preparations and values were shown as mean ± SE (*n* = 8). Asterisk denoted significant differences from controls (Student’s *t*-test, “*” denotes *p* < 0.05). Vg protein levels were detected by western blot, an antibody against β-actin was used as a loading control.

To ascertain the effect of AAs as a stimulus activating Vg synthesis in the *N. lugens* females, we used an established chemically defined artificial diet culture system for the insect rearing. Newly emerged females (within 12 h) were reared on artificial diets either containing (+AAs) or lacking AAs (−AAs). In conjunction with the artificial culture system, qRT-PCR was used to determine *Vg* mRNA levels and western blot was applied to measure Vg protein levels in the fat bodies in response to various AAs treatments. As shown in Figure 3B, females treated with depleted AAs for three days (AD3d) demonstrated an approximate 90% reduction in *Vg* mRNA level as compared with the control (AF3d, reared on AAs for three days). Vg protein was not present at a detectable level in the AAs-deprived females, and a large amount of Vg was present in the AF3d control group. To determine whether AAs have any activating Vg expression effect, females lacking AAs in the first and second day (AD2d) were supplied with AAs in the third day (AR3d). Supplement of AAs yielded a significant increase in *Vg* mRNA levels, about a 4.5-fold higher level as compared with the females in absence of AAs continuously (AD2d). Similarly, a considerable amount of Vg protein was present after a supplement with AAs (Figure 3B).

### 2.4. JmtN Expression Is Regulated by AAs Signaling

**Figure 4:**
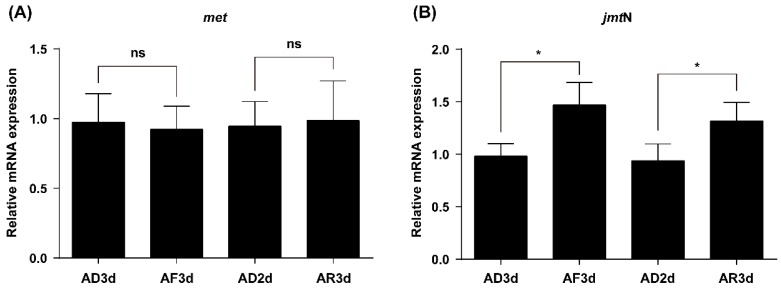
Expression of *met* (**A**) and *jmt*N (**B**) mRNA in AAs-deprived females. Newly emerged females (within 12 h) were reared on artificial diets either containing (+AAs) or lacking amino acids (−AAs). Relative mRNA expression levels of *met* in the FB and *jmt*N in the CA were detected using qRT-PCR. Bars represent mean ± SE of 7–8 biologically independent pools of 30–50 females and three technical replicates, normalized with *β-actin* (*n* = 7–8, Student’s *t*-test, “ns” denotes *p* > 0.05 and “*” denotes *p* < 0.05).

To determine the effect of AAs on transcription of the *met* and *jmt*N in the *N. lugens* females, the relative mRNA expression levels between the AAs-fed and deprived females were analyzed by qRT-PCR. The mRNA expression levels of *met* in the fat body remained constant without significant differences among the different AAs treatments (Figure 4A). However, the *jmt*N mRNA level in the corpora allata significantly dropped in the AAs-deprived females (AD3d) compared with their mRNA expression levels in these AAs-fed females (AF3d). Furthermore, *jmt*N expression was successfully induced after AAs supplement (AR3d) compared with its expression in the AAs-deprived females (AD2d) (Figure 4B).

### 2.5. JH Mediates AAs Signaling that Regulates Vg Synthesis

**Figure 5:**
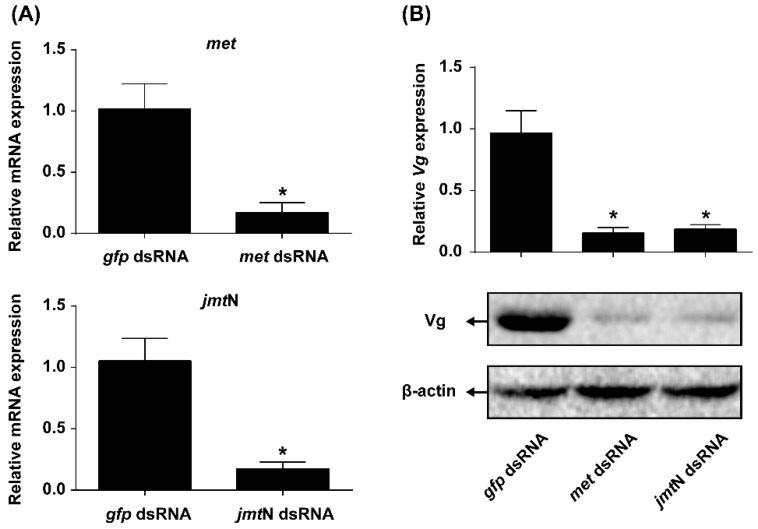
Knockdown of *met* or *jmt*N by RNAi results in significant downregulation of Vg induced by AAs. (**A**) Knockdown efficiency of *met* and *jmt*N by RNAi. Relative mRNA expression levels of *met* in the FB and *jmt*N in the CA in *gfp* dsRNA control females were set to 1; (**B**) Effect of the JH pathway genes knockdown on the mRNA and protein levels of Vg. Total RNA and protein were isolated from fat bodies of females. *Vg* transcript levels were detected in comparison to *β-actin* mRNA levels by qRT-PCR. The expression levels of *Vg* transcripts in each RNAi treatment were determined by setting the control insects (*gfp* dsRNA) *Vg* transcript expression levels at 1. Values were shown as mean ± SE of seven independent replications (*n* = 7). Asterisk denotes significant differences from controls (Student’s *t*-test, “*” denotes *p* < 0.05). Vg levels were analyzed by western blot and *β-actin* was used as a loading control.

To investigate whether JH is required for AAs-induced Vg synthesis or not, expression of the genes coding for key proteins involved in JH biosynthesis (JHAMT) and JH receptor (Met) were knocked down by injecting respective dsRNAs. The knockdown efficiency of *met* and *jmt*N was 83.13% and 83.28%, respectively (Figure 5A). Knockdown in the expression of *met* or *jmt*N resulted in about 80% reduction of *Vg* mRNA compared with the females treated with *gfp* dsRNA. Furthermore, in *met* or *jmt*N dsRNA treated females, Vg levels were many fold lower in response to stimulation by AAs as compared with Vg levels in females injected with *gfp* dsRNA (Figure 5B). 

**Figure 6:**
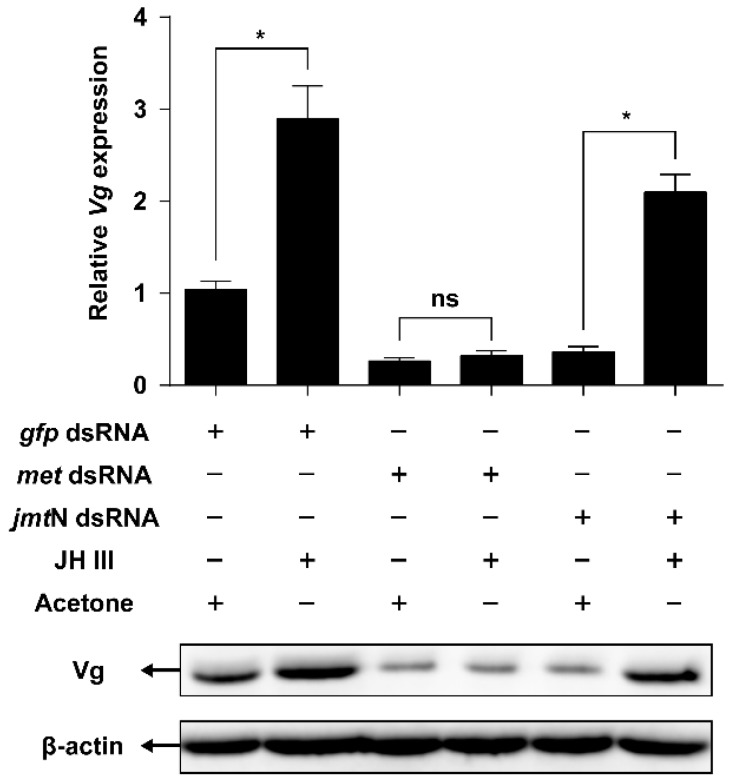
Effect of JH III on AAs-induced Vg synthesis. Newly emerged females were injected with 100 ng of respective dsRNA targeting *met*, *jmt*N or a nonspecific *gfp* and reared for two days. After that, the insects were topically applied with JH III (100 ng in 100 nL acetone) or acetone (100 nL) and fed with artificial diets for another day. Relative mRNA levels of Vg in the FB were detected by qRT-PCR (*n* = 7, Student’s *t*-test, “ns” denotes *p* > 0.05 and “*” denotes *p* < 0.05). Vg protein levels were detected by western blot.

JH III was applied to the females injected with *met* or *jmt*N dsRNAs. Application of JH III rescued *Vg* mRNA and protein levels in *jmt*N dsRNA-injected females. However, knockdown in the expression of *met* prevented the ability of JH III to restore the expression of the *Vg* gene to the same protein level found after injection of dsRNA of *gfp* or *jmt*N (Figure 6).

### 2.6. Silencing Met or JmtN Inhibits Ovarian Development and Reduction in Fecundity

**Figure 7:**
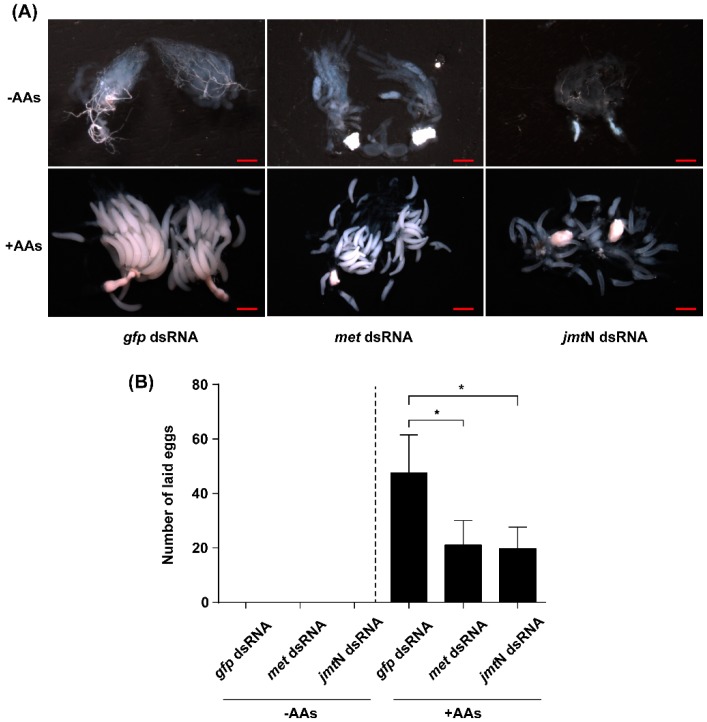
Ovarian growth and fecundity in the RNAi, AAs-deprived and fed females. (**A**) AAs, *met* and *jmt*N are required for ovary development. Equal amount of dsRNA for JH pathway genes and *gfp* (control) was injected into newly emerged females (within 12 h). The ovaries were dissected on day 6 post adult emergence and photographed with stereo microscopy SMZ18 (Nikon, Tokyo, Japan). Scale bars indicate 500 μm; (**B**) Fecundity was reduced in *met* and *jmt*N dsRNA-treated females. The total number of eggs laid was counted for fifteen days. Since AAs-deprived females laid no eggs, only egg numbers derived from AAs-fed females were used for statistical test. Bars represent mean ± SE of three independent experiments of ten females and asterisk indicates significant differences (Student's *t*-test, “*” denotes *p* < 0.05).

To investigate the role of JH and AAs on ovarian development and fecundity, newly emerged females were injected with dsRNA of *met*, *jmt*N or *gfp* and reared on an artificial diet. Ovaries were dissected at day six from control and dsRNA injected females, and photographed using a stereo microscope. The development of ovaries that were dissected from AAs-deprived females was completely blocked, and no mature eggs were found. In the AAs-fed group, fully developed eggs and matured ovaries were observed in the *gfp* dsRNA-injected controls. However, knockdown of *met* and *jmt*N transcript levels by dsRNA severely inhibited the ovarian growth and oocyte maturation (Figure 7A). Those females that were injected with *met* or *jmt*N dsRNA and reared on normal diet (+AAs) laid fewer eggs, with an average of 21.1 and 19.8 per female respectively, a significant decrease (55.67% and 58.4%, respectively) as compared with the number of eggs laid by the control group (injected with *gfp* dsRNA), and no eggs were laid by the AAs-deprived females (Figure 7B).

## 3. Discussion

The effects of nutrition on female reproduction have been demonstrated in anautogenous mosquitoes and some other insect species [30,31,32]. JH regulation of female reproduction, especially vitellogenesis and egg development, has also been demonstrated in many insects [33,34,35]. Moreover, JH is also involved in the nutrient-dependent regulation of insect reproduction, but the mechanism of action remains obscure. Previous studies have shown that JH III is the major hormone regulating Vg synthesis in Hemiptera [36], while 20-hydroxyecdysone (20E) and JH activate the *Vg* gene in Diptera [5,22]. There exists only a single copy of the *Vg* gene in *N. lugens* and JH III not only stimulates *Vg* expression in the fat body [21], but also regulates its uptake by the developing oocytes in *N. lugens* [25]. 

In this study, we first identified two genes coding for key proteins in the JH biosynthesis (JHAMT) and JH receptor (Met) (Figure 1). JHAMT, a terminal enzyme that is involved in the JH biosynthesis, has been shown to be required for the expression of the *Vg* gene [15,19]. In the cockroach *Diploptera punctate*, RNAi-mediated silencing of *Met* severely inhibited ovarian growth and caused a significant decrease in the size of developing eggs [37,38]. Our results showed that in *N. lugens* the control of vitellogenesis and ovarian growth by nutrition is mediated by JH biosynthesis and does not appear to work through regulation of the JH receptor as *jmt*N expression levels in the CA were significantly elevated by AAs, while *met* mRNA levels in the fat body remained constant (Figure 4). This is not surprising because receptors are fairly stable and do not fluctuate once they are synthesized, whereas enzymes are up and down regulated depending on whether the cell need the enzyme for synthesis of JH III [37]. In *T. castaneum*, the expression of *jmt*N was stable after starvation treatment [19], which is different from that in *N. lugens*. To examine the role of JH in AAs-mediated Vg synthesis, we performed RNAi experiments. As expected, RNAi-mediated depletion of *jmt*N or *met* seriously blocked *Vg* gene expression and Vg protein synthesis (Figure 5B), suggesting that the AAs are needed for Vg biosynthesis and may release JH but not necessarily affect *jmt*N. The regulation of vitellogenesis by exogenous application of JH or JH analogues has been shown in many insect species and also confirmed by our previous experiments [15,25]. Methoprene application on *met*-depleted locusts failed to restore *Vg* expression to normal levels, indicating that *met* mediates the induction of *Vg* expression by exogenous application of JH analogues [20]. To further confirm the significant role of JH in the regulation of AAs-induced Vg synthesis, we applied exogenous hormone. In our experiments, the addition of JH III can overcome the lack of JHAMT, however, once the JH receptor is blocked JH cannot be an effective hormone without binding to its receptor (Figure 6). Also it is not surprising that JH can be substituted for JHAMT after the *jmt*N was knocked down by dsRNA, because it is the last step in the JH III biosynthetic pathway [29], whereas the JH receptor Met cannot be substituted with JH, because the receptor is needed for the JH to bind to it and then activate the *Vg* gene [37]. So these are two different mechanisms and JH was never shown to activate the Met receptor only to bind to it. Detailed studies in the mosquito, *A. aegypti* and the cockroach, *B. germanica*, revealed that insulin-like peptides (ILPs) regulate JH biosynthetic activity of the CA, thus placing JH downstream of the insulin signaling [13,39]. Perhaps Met is also activated by ILPs or even by other hormones, further studies are needed to confirm the possibility. Our results suggest that AAs induce *Vg* gene expression in the planthopper, *N. lugens*, by releasing JH or other hormones that stimulate the synthesis of JH like allatotropin, rather than through JH action. Conversely, in an endoparastic wasp, *Pteromalus puparum*, application of 20E induced *Vg* gene expression while JH decreased the mRNA levels of *Vg* [40]. In the beetle *T. castaneum*, JH controls *Vg* gene expression in the fat body and 20E regulates Vg synthesis through its influence on ovarian development and oocyte maturation [15,41]. In mosquitoes, both JH and 20E have a role after the blood meal as was shown by Borovsky *et al.* [22].

JH regulation of Vg synthesis is complex, both direct and indirect regulatory mechanisms were reported. For direct effect, the *Vg* gene expression depends on the binding of a hormone-receptor complex to hormone responsive elements (HREs) usually located in the promoter region of the genes [42]. The analysis of insect *Vg* sequences suggested the presence of juvenile hormone responsive elements (JHREs) in the *Vg* promoter regions [36,43,44]. Recently, the promoter sequence of *Vg* was identified in *N. lugens* [45]. Furthermore, a number of DNA elements that match with consensus JHREs were found in the promoter region of the *N. lugens Vg* gene, suggesting that *Vg* is a JH responsive gene. On the other hand, JH regulates the expression of ILPs that in turn induces Vg synthesis in *T. castaneum* [19]. The regulation of Vg synthesis by nutrient-sensing insulin/insulin-like growth factor (IGF) signaling (IIS) and target of rapamycin (TOR) pathways has been reported in mosquitoes, honey bees, fruit flies, cockroach and beetles [1,12,30,46,47]. Silencing of the forkhead box transcription factor O (FoXO), the main transcriptional effector of the IIS pathway, or injection of bovine insulin into JH-deficient or starved *T. castaneum* females, induced Vg synthesis, suggesting the possible cross-talk between JH and IIS in regulation of vitellogenesis [19]. However, knockdown of *FoXO* in starved *B. germanica* females could not fully restore the JH and *Vg* mRNA levels, implicating that some other regulatory factors may be involved in this process [39]. More recently, the key components of IIS and TOR pathways were identified in *N. lugens* [48,49]. Interestingly, it has been demonstrated that *N. lugens* wing morph determination is controlled through the IIS pathway [49]. Our data presented here clearly demonstrates that AAs regulate Vg synthesis by inducing JH biosynthesis in *N. lugens*. The cross-talk among JH, TOR and IIS pathways in this nutritionally dependent reproductive process needs further experimentation.

## 4. Experimental Section

### 4.1. Insect Rearing

The *N. lugens* colony was maintained at 26 ± 1 °C, 95% relative humidity and a light:dark (16 h:8 h) photoperiod. Insects were fed on fresh rice seedlings in our laboratory as described previously [25]. One-day-old females were collected and fed on normal artificial diets containing AAs (+AAs) or AAs-deficient diets (−AAs), and artificial diets were prepared according to the protocols provided by Fu *et al.* [50]. Artificial diets were held between two layers of Parafilm “M” and daily replenished.

### 4.2. Sequence and Phylogenetic Analysis

The nucleotide sequence of *met* gene (KJ690934) was obtained from NCBI. The sequence of *jmt*A (NM_001162779) was used to identify the *N. lugens* ortholog in the transcriptome and genome databases using the Blastn algorithm. The amino acid sequences of Met from 16 other insect species and JHAMT sequences from 23 insects were identified by Blastp searches to GenBank. Amino acid sequences of Met or JHAMT were first aligned by ClustW software separately, and the phylogenetic trees were constructed using MEGA 6 software with maximum likelihood method [51].

### 4.3. Tissue Collection

*N. lugens* adult females were dissected in precooled PBS solution (140 mM NaCl (Sinopharm Chemical Reagent Co., Ltd., Shanghai, China), 2.7 mM KCl (Sinopharm Chemical Reagent Co., Ltd., Shanghai, China), 10 mM Na_2_HPO_4_ (Sinopharm Chemical Reagent Co., Ltd., Shanghai, China) and 1.8 mM KH_2_PO_4_ (Sinopharm Chemical Reagent Co., Ltd., Shanghai, China); pH 7.4) using a SMZ745 binocular microscope (Nikon, Tokyo, Japan). For tissue distribution of *met* and *jmt*N, the following tissues were dissected from eight independent pools of 30–50 three-day-old adult females each: BR: brain; CA: corpora allata; FB: fat body; OV: ovary; MG: Malpighian tubule; EP: epidermis. Dissected tissues were immediately transferred to liquid nitrogen to prevent RNA degradation and stored at −80 °C until further processing.

### 4.4. RNA Extraction and cDNA Synthesis

Pooled tissues were homogenized with RNase-free pestles (Tiangen, Beijing, China) and total RNA was extracted using Trizol reagent (Invitrogen, Carlsbad, CA, USA) according to the instructions of manufacturer. The concentration and purity of RNA sample were measured using a Nanodrop 2000C spectrophotometer (Thermo Fisher Scientific, West Palm Beach, FL, USA). DNase treatment was performed to eliminate potential genomics DNA contamination. The first-strand cDNA was synthesized from an equal amount of RNA using a PrimeScript RT Reagent Kit with gDNA Eraser (TaKaRa, Tokyo, Japan) in a final volume of 20 μL following the manufacturer’s instructions.

### 4.5. Reverse Transcriptase Quantitative Real-Time PCR (qRT-PCR)

Primers used for qRT-PCR were designed using Primer3 software (http://primer3.ut.ee/) [52], and the primers were shown in Table 1. Primer sets were validated by generating relative standard curves for each gene transcript using a 10-fold serial dilution of cDNA sample. Amplification efficiency (*E*) and correlation coefficient (*R*^2^) were calculated for each primer pair according to the standard curves. All qPCR reactions were performed in triplicate in 384-well plates on a Light Cycler 480 system (Roche Diagnostics, Basel, Switzerland). Each 10 μL reaction contained 5 μL 2× SYBR Premix^EX^ Taq II Master Mix (TaKaRa, Tokyo, Japan), 0.4 μL forward primer (10 μM), 0.4 μL reverse primer (10 μM), 1 μL cDNA template and 3.2 μL of deionized water. A three-step qPCR was used to quantify gene expression levels in this study with the thermal cycling profiles: 95 °C for 30 s, followed by 40 cycles of 95 °C for 5 s, 60 °C for 15 s and 72 °C for 20 s. Finally, a melt curve analysis was performed to assess amplification specificity. The relative mRNA expression levels of target genes were quantified using the 2^−ΔΔ*C*t^ method [53].

### 4.6. Western Blot

Fat bodies were homogenized in precooled lysis buffer (8 M urea (Amersco, Solon, OH, USA), 4% CHAPS (Amersco, Solon, OH, USA), 40 mM Tris (Amersco, Solon, Ohio, USA) pH 8.0, 5 mM EDTA (Amersco, Solon, OH, USA), 1 mM PMSF (Amersco, Solon, OH, USA), 10 mM DTT (Roche Diagnostics, Basel, Switzerland)) and 0.2 mM protease inhibitor (Roche Diagnostics, Basel, Switzerland), placed at 4 °C for 1 h and centrifuged at 12,000× *g* at 4 °C for 20 min to remove debris. Protein concentration was detected using a Bradford protein assay kit (Tiangen, Beijing, China). A total of 30 μg protein was separated by SDS-PAGE, electroblotted onto polyvinylidene fluoride (PVDF) membrane (Millipore, Billerica, MA, USA), and blocked in 5% nonfat milk (Amersco, Solon, OH, USA) at room temperature for 1 h. After washing with TBST (Tris-buffered saline, pH 7.4, 0.5% Tween-20) (Amersco, Solon, OH, USA), the membranes were incubated overnight at 4 °C with primary antibodies for Vg and β-actin [25]. After washing, the membranes were incubated with goat anti-rabbit immunoglobulin G horseradish peroxidase-conjugated secondary antibody (Sigma, St. Louis, MO, USA) for 1 h at room temperature. The immunoreactivity was detected using SuperSignal West Pico Chemiluminescent Substrate ECL (Pierce, Rockfod, IL, USA) and the membrane was imaged with a GBOX-Chemi XT4 Imager (Syngene, Cambridge, UK). Vg protein expression was compared with the internal control β-actin.

### 4.7. Synthesis and Injection of dsRNA

The templates for *met* (nucleotides 923–1421, 499 bp), *jmt*N (nucleotides 154–701, 548 bp) and the negative control *gfp* (nucleotides 1760–2301, 542 bp) (ACY56286) [54] dsRNA synthesis were generated by RT-PCR with primers containing T7 polymerase promoter sequences (Table 1) and the PCR amplification reactions consisted of 1 cycle of 95 °C for 5 min; 35 cycles of 95 °C for 30 s, 55 °C for 30 s and 72 °C for 1 min; and a final extension step of 72 °C for 10 min. The PCR products were verified by sequencing and used to synthesize dsRNA with a T7 RiboMAX™ Express RNAi System (Promega, Madison, WI, USA). Two complementary RNA transcripts for each gene were generated at 37 °C for 4 h and dsRNA was formed after annealing. The dsRNAs were further purified and suspended in diethyl pyrocarbonate (DEPC)-treated water. Concentration and purity of dsRNA were determined with a Nanodrop 2000C spectrophotometer (Thermo Fisher Scientific, West Palm Beach, FL, USA). The final concentration of dsRNA was adjusted to 4.5 μg/μL with DEPC-treated water. Before dsRNA injection, carbon dioxide was used to anesthetize insects and each newly emerged female was injected with 23 nL dsRNA (about 100 ng) using a Nanoject II microinjection device (Drummond scientific, Broomall, PA, USA) [55]. Control groups were either injected with equivalent volume (23 nL) of DEPC-treated water or *gfp* dsRNA.

### 4.8. Ovarian Growth and Fecundity Analysis

Equal amount of dsRNA for *met*, *jmt*N or *gfp* was injected into newly emerged females (within 12 h) separately. Injected females were placed in an artificial feeding device and reared on artificial diets either containing AAs (+AAs) or lacking AAs (−AAs). The ovaries were dissected in precooled PBS solution on day 6 post adult emergence and photographed with a stereo microscopy SMZ18 (Nikon, Tokyo, Japan). At least fifteen females were dissected in each replicate, and each treatment was replicated three times. For fecundity analysis, each injected female was matched with two males and placed in an oviposition device. Artificial diet was held between two layers of stretched Parafilm “M” at one end of the glass chamber and oviposition medium (5% sucrose with 4 mM salicylic acid) was held on another end of glass cylinder [56]. The artificial diet was replenished daily and the laid eggs were recorded daily for fifteen days. At least ten females were analyzed in each replicate and three independent experiments were performed.

### 4.9. Statistical Analysis

All data are presented as mean ± SE and *Shapiro-Wilk* normality test was used to examine the normality of data variances. A one-way ANOVA followed by Tukey’s honestly significant difference (HSD) multiple comparison test was performed to analyze the tissue-specific expression profiles of *met* and *jmt*N. Data of other gene expression levels and average laid egg numbers was analyzed using Student’s *t*-test. All statistical analysis was performed with SPSS 18.0 software (SPSS Inc., Chicago, IL, USA) and differences between the different groups were considered significant at *p* < 0.05.

## 5. Conclusions

In summary, the whole results indicate that AAs are indispensable for the Vg synthesis and egg development in *Nilaparvata lugens*, and suggest that AAs induce *Vg* expression by activating JH biosynthesis, rather than through JH action.

## Figures and Tables

**Table 1:** PCR primers used for qRT-PCR and dsRNA synthesis.

Purpose	Primer Name	Primer Sequence (5′–3′)
**qPCR analysis**	Q-*met*-F	AGTGGCAGCGAGCGATGATT
	Q-*met*-R	TGAGGCGCAGCAAAAAGGAG
	Q-*jmt*N-F	GAACCTGCAGGCCAAACACA
	Q-*jmt*N-R	ACCACTCGGTTGGGCTGAAT
**dsRNA synthesis**	*met*-Fi	GACCCAAGCCACCCTCCAAG
	*met*-Ri	TCCCCATCGTCAGCCAACTC
	*met*-T7Fi	taatacgactcactatagggGACCCAAGCCACCCTCCAAG
	*met*-T7Ri	taatacgactcactatagggTCCCCATCGTCAGCCAACTC
	*jmt*N-Fi	CTCCAGGCCATTGTCCCTCA
	*jmt*N-Ri	TTGGCCTGCAGGTTCTTTGG
	*jmt*N-T7Fi	taatacgactcactatagggCTCCAGGCCATTGTCCCTCA
	*jmt*N-T7Ri	taatacgactcactatagggTTGGCCTGCAGGTTCTTTGG

F: Forward; R: Reverse. Lowercase letters labeled sequences represent for T7 promoter.

## Supplementary Files

Supplementary File 1
