# Supplementary figures and images for: Nutritional Signaling Regulates Vitellogenin Synthesis and Egg Development through Juvenile Hormone in Nilaparvata lugens (Stål)
Source: Int J Mol Sci. 2016 Feb 26;17(3):269. doi: 10.3390/ijms17030269 (PMC4813133; doi:10.3390/ijms17030269)

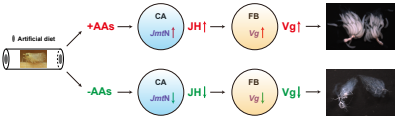

Supplement: Supplementary File 1 [file ijms-17-00269-s001.pdf]
